# Helping traumatized people survive: a psychoanalytic intervention in a contaminated site

**DOI:** 10.3389/fpsyg.2014.01419

**Published:** 2014-12-08

**Authors:** Fanny Guglielmucci, Isabella G. Franzoi, Chiara P. Barbasio, Francesca V. Borgogno, Antonella Granieri

**Affiliations:** Department of Psychology, University of TurinTurin, Italy

**Keywords:** collective trauma, contaminated site, basic trust, psychoanalytic group, mourning

## Abstract

Psychoanalytic literature on extreme traumatization usually distinguishes between natural catastrophes and man-made catastrophes. While the first ones are usually sensed as nature’s ferocity, fate, or God’s will, the second ones are experienced as a volountary and violent attack aimed at disrupting other human beings. In this paper we focus on man-made disasters caused by a profit-driven logic. When traumatization is due to irresponsible actions perpetrated by the owners of the major economic resource of a community, it deeply affects the identity of the group, entailing the loss of basic trust and lively parts of the Self. In such a situation, where the whole community is severely traumatized, psychoanalytic group therapy seems to be the most suitable setting: it allows to place the historization of the event and the creation of multiple narratives of somato-psychic suffering. Trust and faith are two crucial factors in the encounter with patients lacking a sense of vitality. The working through of each one through the group field is an essential forerunner to the construction of a recovered sense of faith and reliability that precedes the onset of a true new-beginning.

Psychoanalytic literature on large scale disasters and extreme traumatization usually distinguishes between accidents or natural disasters (e.g., hurricanes, earthquakes, etc.) and man-made traumatizations (e.g., rape, torture, war, terrorism, etc.; de Dunayevich and Puget, 1989; Varvin, 1995, 2003, 2005; Volkan, 2001; Wusmer, 2004; Rosenbaum and Varvin, 2007; Boulanger et al., 2013). If the first category is generally felt as nature’s ferocity, fate or God’s will, the second one is experienced as a volountary and violent attack aimed at disrupting and destabilizing specific groups and communities. Volkan underlines that even if both cause grief, anxiety and massive traumatization:

“After man-made accidental disasters, survivors may blame a small number of individuals or governmental organizations [but] there are no ‘others’ who have intentionally sought to hurt the victims. When […] there is an identifiable enemy group who has deliberately inflicted pain, suffering, and helplessness [such a] trauma affects large-group (i.e., ethnic, national, or religious) identity issues in ways entirely different” (Volkan, 2004, p. 481).

This is certainly true for natural disasters such as hurricane Katrina in 2005, or the massive earthquake in Turkey in 1999, as well as for accidental man-made disasters such as the Chernobyl nuclear catastrophy in 1986.

But what happens when an entire community is victim of a profit-driven logic? When the ‘others’ who have caused illness and death with their careless decisions and irresponsible behavior are the major economic resource of the community? Can we still consider this ‘accidental’?

Our research in Casale Monferrato – an Italian town considered the capital of asbestos-cement production for almost 80 years – has shown that living in a contaminated site (CS) may have important psychological consequences. Although today the production of asbestos is forbidden, the inhalation of its fibers has led to more than 3000 victims in this community over the years. The illness has affected not only the workers but the whole population (Fazzo et al., 2012). Moreover, people who live there are still at risk: the malignant pleural mesothelioma, a rare and fatal lung cancer caused by the inhalation of asbestos, has an incubation period of about 30 years. This means that in the next two decades the mesothelioma will kill at least 500 more persons (Furlan and Mortarino, 2012; Italian Ministry of Health, 2012). From a psychoanalytical perspective, living with fear of ‘aerial contagion’ by an ‘invisible killer’ produces post-traumatic conditions: the population shows depressive symptoms, high levels of anxiety, a tendendency to exteriorization and to somatizatize affects, dissociative experiences and long-lasting adverse effects in the personality (Granieri, 2008, 2013; Granieri et al., 2013). These are the same conditions observed in those populations exposed to Chernobyl’s nuclear radiations (Loganovsky et al., 2008; Bromet et al., 2011). In both circumstances the exposure to a pathogen has represented a traumatic event that has aroused catastrophic affects, thus entailing the loss of healthy aspects of the Self.

Nevertheless, we think that the quality of this two experiences is profoundly different. According to Varvin (2013, personal communication) when illness and death are caused by the major economic resource of a community – for example Katowice in Poland, the ‘Terra dei Fuochi’ and Casale Monferrato in Italy, Stuttgart in Germany – this adds an experiential quality that worsens the situation. When a whole population is exposed to death and many people are killed in the name of a ‘higher cause’ (religion, justice, but also money, and power) a certain responsibility and intentionality can be traced. This kind of trauma affects the identity of the large-group that has progressively been built around the idealization of a powerful mother/factory which feeds all her children with toxic food. The unconscious life of CS’ communities is characterized by primitive mechanisms of denial and splitting: only these allow the people to continue living there without experiencing fragmentation anxieties and overwhelming feelings of shame and guilt connected to the fact that they have accepted something dangerous and deadly for themselves and their families.

In such a situation the Ego is forced to undertake the evaluation of a contradictory reality and this leads therefore to a detachment among some aspects of the “*narcissistic contract*” (Aulagnier, 1975) between the individual and the society. Little by little the social context becomes incomprehensible and ungraspable and people feel betrayed and violated: the basic assumption that the world is a safe and orderly place is profoundly damaged and the basic trust starts to waver. Unconscious conflicts between life and death, economic prosperity, and mourning take place, opening the field to helplessness, hopelessness, and shared aggressive fantasies directed toward the source of the trauma: the factory.

Under these circumstances the population may react in two different ways:

1. Social adaptation: the violent irruption of the trauma alters the ability to use critical thinking and alarm mechanisms, leading to collusion with the account provided by the factory. Through a defensive mimetic attitude, subjects adapt to a traumatic external reality and become then able to ‘tolerate’ its threatening and unbearable aspects.
2. Defensive cohesiveness: some citizens gather into Unions and Associations joining efforts in legal battles against those who have committed the ‘community murder’ (see for example the legal battle in Casale Monferrato ^1^). The factory becomes thus a common enemy, while subjects become fighters for a ‘right cause’.

Beyond these regressive and defensive stances we can trace overwhelming and catastrophic affects that “*cause destabilization in the capacity for symbolizing emotional experience including giving meaning to the life after the trauma*” (Rosenbaum and Varvin, 2007, p. 1528). The impossibility to signify pain and to symbolize deadly experiences also compromises the ability to mourn the loss, leading the community to a “*perennial mourning”* (Volkan, 2001), a psychic state characterized by a frozen internal life and a libidinal disinvestment in the inner world. But there is something more: when the whole community has to face a massive trauma, regression reflects the efforts of the group to maintain, protect, modify, or repair the shared group identity (Volkan, 2002).

In our clinical practice with the traumatized population of Casale Monferrato we have encountered many citizens who could define their identity only through their belonging to the factory and the deaths caused by it. For most of them the question “*who am I?*” has been replaced by the question “*whom have I lost*?”

Giulia: “This damned factory has always been here since I can remember. It fed all of us for more than 50 years, but at what cost? Everyone here is a victim of the factory […] Everyone has lost a loved one and everyone is going to die because of the asbestos dust that is still in the air”

We have traced this deep pain and rage for the losses caused by a meaningless reason and a deep fear to die and lose dear ones again in many others subjects who live there (Granieri and Borgogno, 2014).

How can psychoanalysis help CS’s communities to survive these shared traumas?

Exploring mental group life and recognizing the importance of the actual environment in which citizens have lived and live are essential factors in this process. The “*elasticity of technique”* (Ferenczi, 1928), the ability to move “beyond the couch” and explore the destructive impact of a social catastrophe – keeping in mind that the whole community is still immersed in a traumatizing environment – has been a necessary element without which our analytic journey would not have been truly analytic.

As far as we know, there is no evidence of group interventions in CSs. Group interventions for PTSD patients are mostly cognitive (Fallot and Harris, 2002; Cox et al., 2007; King et al., 2013) or psycho-education programs (Kiser et al., 2010; Sherman et al., 2011; Shumway et al., 2011; Fischer et al., 2013). Differently from these perspectives, we think that a the psychoanalytic group represents “*a privileged space for a certain type of thinking, of recognition and transformation of suffering, the fear of losing the social identity of being a victim […] and being a relative of a [murdered] person”* (de Dunayevich and Puget, 1989, pp. 110–111).

In this perspective, two of the authors (Antonella Granieri and Francesca V. Borgogno) have conducted for almost two years a multifamily group (García Badaracco, 1989, 2000) promoted by oncologists, palliativists, and general practitioners, as well as by the Association of Families and Asbestos Victims, the Local Public Authority, and the media. In accordance with the original setting proposed in Argentina by Garcìa Badaracco for severe psychiatric disorders, it met once per week and was open to whoever wished to participate: patients, relatives, health, and social practitioners, and in general to any citizen who was interested. Each session lasted ninety minutes and was followed by thirty minutes of working through carried out by the therapists alone ^2^. Although our group maintained the same setting of the original one, points of convergence and divergence emerged in our clinical practice. As the latter, in our setting group and family dimensions together gave birth to a “*mini-society*” that allowed the development of the “*healthy vitality*” of the patient (Borgogno, 2010). However, we have to underline that psychiatric patients who are unable to separate themselves from their families are profoundly dissimilar from mesothelioma patients, who are instead obliged to separate from their loved ones in a very short time. Moreover, the main theme of the group was quite different: in Casale this was often death, which revealed the presence in both patients and their families of deep deadly anxieties as a consequence of living in a “*murdered environment.*”

During the years about 50 people participated to our group: most of them were relatives, some patients, while the participation of health professionals was occasional because of “*emergencies in clinical practice.*” Such a behavior seemed to underline health professionals’ difficulties in separating themselves from the task of taking care only of the physical consequences of the disease and it was important to explore in the group the fantasies connected with this unexpected limited participation (i.e., the feeling that health professionals were not so interested in the psychological work of the group considered as “*not important*”), reconnecting such a behavior to a defensive avoidance of psychological pain that circulate in the group.

As García Badaracco (1989, 2000) pointed out, the group works as a sort a of “*extended mind*” where each individual contribution stimulates the potential of the group generating free associations in a continuous game of reciprocal identifications. In such a situation, transferences and counter-transferences are not developed around one person but they are multiple and are dispersed on therapists and other members of the group, both objects of the transference. Working with patients with such a short life expectancy raised countertransferal intense feelings of helplessness and the co-leaders sometimes felt that their work was threatened by the impending death, omnipresent in the analytic encounters. The possibility to ‘dilute’ transferences and counter-transferences in the group, making these feelings more bearable and expressible in different words by different participants, representing thus a very significant therapeutic factor (Granieri and Borgogno, 2014) ^3^.

In the group, we worked with survivors helping them symbolize and mentalize the mourn, the deep guilt for not sharing the ill-fated destiny of a loved one and the intense anger directed towards the factory and the Government, who “*didn’t do enough*” to protect the citizens. Taking part into the group made it possible, in time, to tell someone the pain and the anguish one is feeling, without being ashamed. During the sessions it became gradually possible to create a field where thinking to and digesting traumatic emotions (Granieri and Schimmenti, 2014), and integrating dissociated parts of the Self (Schimmenti and Caretti, 2014). In this sense, the group can be thought of as a kind of “*cradle*” where emotions can see the light again, a space where it was possible to put into words non-mentalized aggregates. Identifying and recognizing these unconscious and embodied feelings was a necessary step in order to promote a genuinely analytic and transformative process (Seganti et al., 2003; Granieri, 2011a,b).

At the beginning, this community found it really difficult to remember its life *before* the trauma and could not think a life *beyond* it.

Vincenzo: “I worked there for almost 25 years, no one told us it was dangerous, but they knew it, they all knew it. Everyone said it was safe and that I was lucky to work there because it was a well-paid job. I didn’t know I would get sick because of my job and probably I will die for it!”

Obviously living in a world where things are not how they are told to be has deeply compromised the sense of safety, opening the door to unthinkable frightening deadly experiences.

The core question is: how is it possible to bring into light lively and vital parts in a population immersed in death?

We suggest it can be fostered through trust and faith. According to Neri (2005) these two factors are crucial when we meet patients lacking a sense of vitality. Trust originates from lasting and reliable relationships which promote the idea that the world is a safe and predictable place; while faith is a sort of ‘driven force,’ “*the result of ‘internal work.’ [It] could be seen as the outcome of a series of experiences and situations where we have trusted somebody or something because they have responded consistently to our expectations and needs*” (Neri, 2005, p. 82).

In the group it has been necessary to explore feelings involved in having trust and faith. People asked us to contain and mitigate catastrophic affects and the perception of a threatening, persecutory, and unpredictable world. At the same time, they wished for us to become an object capable of supporting their potential for growth, transmitting them the hope that it was possible to psychologically survive the trauma despite the impossibility to change a deadly past and destiny.

Patients and survivors needed to be “*understood in having value and existing for another person who affectively and mentally participates in their particular experiences*” (Borgogno, 2013, p. 28). In the “*long wave*” (Borgogno, 2007) of the work with the group, we learnt to work with the people who were there and with the empty chairs: the ghosts of the dead, lively trace in the mind. The advantage of the possibility of “*feeling with*” (Ferenczi, 1932) has to do with the possibility to get close to the most profound feelings of the other and sharing our desire to work together, an obliged pass in order to build the therapeutic alliance.

Little by little the group has promoted new libidinal investments, allowing the internalization of new emotional nuances and values (vitality, warmth, responsiveness, introspection) presentified in the analytic field. This step was absolutely crucial for reconstructing the sense of belonging and the possibility of sharing common sense compromised by trauma (Bion, 1992). The possibility to invest a new object and to regain the chance to have faith in something or somebody is obtained through an indispensable “*act of credit”* directed not only to our patients and their families, but also towards their potential, “*[managing] to ‘materialize’ (and give proof of) something different*” (Borgogno, 2014, p. 91).

Lia: Though there is a reason why someone who has lost a son is more likely to nominate the son who isn’t here more than the other: the one who is alive is still at our side, he exists.

Antonella Granieri: But he exists and he has the right to exist.

In these traumatic circumstances it is important to have a *sense of perspective*, which patients and families are lacking or have lost. It has been necessary to establish a space between the past and present, to regain the personal history and the capability to live a present and a future less contaminated by the trauma. During the sessions we assisted to a transition to a new involvement in more lively activities.

Lia: When my daughter told me I was to take care of my granddaughter on my own, at first I thought I did not have the necessary energy. But then I thought that meeting people during my walks and the spontaneous exchanges that derive from this activity have always been a resource for me. I thought that that energy is still in me and that I will take Margherita out in the streets of Casale.

This short extract shows the delicacy of the unexpected arousal of a deep joy of life, associated to accompanying her newborn granddaughter through the streets of Casale, a sort of an ongoing presentation of a new generation. We believe the birth of this kind of joyful feelings is strongly connected to the opportunity, offered by the multifamily group, of thinking together about death knocking on each citizen’s door. The possibility to share among several minds the meaning of the trauma has also brought into the field also the vital aspects of each participant, maybe feeble in each individual, but consistent in the functioning of the mind of the group, leading to a new and more mature psychic asset, able to face death and pain.

## Conflict of Interest Statement

The authors declare that the research was conducted in the absence of any commercial or financial relationships that could be construed as a potential conflict of interest.

## Footnotes

1 The appeal sentence condemned the accused who are still alive to 18 years of imprisonment, due to voluntary permanent environmental disaster and voluntary negligence in health and safety measures. The Court also decided that the Region of Piedmont was due to pay a fine of 20 million euros, and the Casale Monferrato City Council a fine of 30.9 million euros.

2 All sessions were audio-recorded and transcribed verbatim, and informed written consent was collected. This paper includes some intervention of group participants, quoted verbatim on the basis of this transcription.

3 In multifamily groups every emotionally charged event or interaction is connected to aspects of the transference to some extent. However, it is not possible to make all of these multiple transferences visible: what is really important is to draw attention to the transference phenomena in front of all participants, so that anyone can discover the dimension of transference, can think about it and see it in others first and then in him/herself (García Badaracco, 2000).
